# Omega-3 polyunsaturated fatty acid profiles and relationship with cardiometabolic risk factors in Cree (*Eeyouch*) of Northern Québec

**DOI:** 10.3402/ijch.v75.30361

**Published:** 2016-07-15

**Authors:** Françoise Proust, Olivia Drescher, Elhadji A. Laouan-Sidi, Elizabeth Robinson, Michel Lucas, Éric Dewailly

**Affiliations:** 1 Population Health and Optimal Health Practices Research Unit, CHU de Québec Research Centre, Québec, QC, Canada; 2 Public Health Department of the James Bay Cree Territory, Montréal, QC, Canada; 3 Department of Social and Preventive Medicine, Université Laval, Québec, QC, Canada

**Keywords:** polyunsaturated fatty acids, traditional food, fish, nutritional transition, James Bay Cree, cardiovascular disease risk factors, lipids, blood glucose

## Abstract

**Background:**

n-3 long-chain polyunsaturated fatty acids (LC-PUFAs) from fish are known modulators of cardiometabolic risk factors.

**Objective:**

To examine fatty acids (FAs) status and the relationship between n-3 LC-PUFA and cardiometabolic risk factors in Cree participants.

**Design:**

We analyzed data from a cross-sectional study (n=829) conducted in Cree adults (aged 18–74 years) from 7 communities of the James Bay territory of Quebec (Canada) in 2005–2009. Sociodemographic, lifestyle, clinical and anthropometric data were collected. FAs were quantified in red blood cells (RBCs) under fasting conditions.

**Results:**

A total of 89% of the participants were overweight (with 69% obesity), 33% had hypertriglyceridemia, 44% had low plasma HDL-c and 77% had fasting plasma insulin ≥90 pmol/l. Total n-3 PUFAs accounted for 6% of total FAs and were higher among older participants, while n-6 PUFAs accounted for 31% of total FAs and were higher among younger participants. According to the adjusted multiple linear regression models, n-3 LC-PUFA was associated (p<0.05) with higher total cholesterol, LDL-c and apo B-100, and was also associated (p<0.05) with lower blood glucose.

**Conclusion:**

Overall, this study showed that n-3 LC-PUFA levels measured in the RBCs of the Cree adults are relatively low and tend towards lower levels among youth. These levels might be insufficient to offset the prevalence of cardiometabolic risk factors.

The traditional diet of James Bay Cree, called *Eeyouch*, was based on local food gathered from fish, game (moose, caribou, bear, and so on), birds (geese and ducks) and wild plants and fruits (berries) (1,2). The *Eeyouch* live in 9 communities distributed in the James Bay territory, called *Eeyou Istchee*, which is located between the 49th and 55th parallels in the province of Quebec (Canada) (Fig. 1), and covers >350,000 km^2^. In 2006, the total population of the James Bay territory was 30,000 inhabitants, half of whom identified themselves as Cree persons (3).

**Fig. 1:**
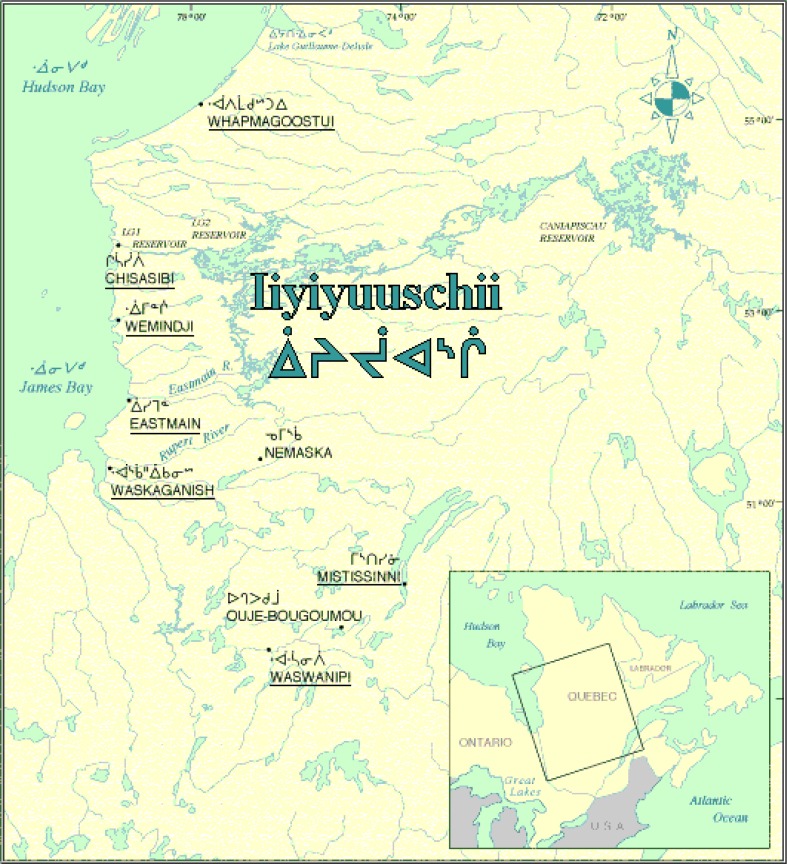
Map of the James Bay territory (*Eeyou Istchee*) (Quebec) and localization of the communities participating in the study.

Much like other indigenous populations, the *Eeyouch* faced rapid cultural and environmental changes associated with reduced reliance on traditional foods and the increased consumption of nutritionally poor market foods (4). This transition, along with diminished physical activity and a more sedendary lifestyle (5), has resulted in an increased prevalence of obesity (4) and chronic diseases (5). Among 850 Cree adults, aged ≥19 years, from 7 of the 9 *Eeyouch* communities, the prevalence of obesity was 71.5% in 2005–2008 (4) and the crude prevalence of type 2 diabetes (T2D) was 17.3%, a 300% increase over the previous 16 years (6).

Fish are naturally rich in omega-3 long-chain polyunsaturated fatty acids (n-3 LC-PUFA), especially eicosapentaenoic acid (EPA, 20:5n-3), docosapentaenoic acid (DPA, 22:5n-3) and docosahexaenoic acid (DHA, 22:6n-3). The measurement of N-3 LC-PUFA in plasma or red blood cells (RBCs) generally reflects habitual fish consumption (7). As opposed to dietary self-reports, these biomarkers provide quantitative measurements that are independent of the subject's memory and/or knowledge (8). The American Heart Association has recognized that fish intake and n-3 LC-PUFA protect against cardiovascular diseases (CVD) (9).

In 1991, the first health survey among the *Eeyouch* of Northern Québec (2) revealed that Cree had higher plasma EPA+DHA (3.9%, 95% confidence interval (CI): 3.8–4.0) and fish intake (60 g/day, 95% CI: 47.5–71.9) compared with Southern Quebecers (EPA+DHA=1.8%, 95% CI: 1.7–1.8; fish intake=13 g/day, 95% CI: 12.7–14.4) (10). The nutritional transition, associated with a shift away from a traditional diet towards retail food, particularly among young people, has resulted in an increased prevalence of cardiometabolic risk factors (11). By keeping some elements of their traditional diet, such as fish rich in n-3 LC-PUFAs, the Cree may attenuate cardiometabolic risks. We therefore analyzed data from the cross-sectional *Eeyou Istchee* study to assess the fatty acids (FAs) status among Cree adults and to determine the relationship between RBC n-3 LC-PUFAs and cardiometabolic risk factors.

## Methods

### Population and study design

The Multi-Community Environment and Health Study, “Nituuchischaayihitaau Aschii,” was conducted between 2005 and 2009 in 7 of the 9 Cree communities located in the *Eeyou Istchee*. The design of this study has been described elsewhere (4,6). Briefly, sampling followed a stratified design based on age categories (0–7, 8–14, 15–39 and ≥40 years). Simple random sampling without replacement was used to select participants within each age stratum in order to build a list of potential study subjects. Local recruiters fluent in *Iiyiyiuyimuwin* (Cree language) and English were responsible for contacting participants by phone and inviting them to enrol in the research project. Local publicity and radio advertising generated and maintained public interest. Of the 2,240 eligible candidates aged 15 years and older, 950 participated voluntarily in the study (42.4%), of whom 853 aged 18–74 years completed clinical and nutrition data. From this sample, 24 subjects did not have FA measurements and were excluded from FA analysis. RBC FAs were therefore analyzed in 829 Cree participants, while 613 subjects were retained for lipid analysis after the exclusion of subjects who did not fast for at least 8 h before blood sampling (n=1) or whose fasting status was not known (n=32), and those taking medication(s) for CVD problems (dyslipidaemia, high blood pressure (HBP) and T2D) at study enrolment (n=183).

Participation was voluntary and subject to written consent. Consent forms were approved by the Comité d’éthique de la recherche de l'Université Laval and accepted by the Research Ethics Boards of McMaster University and McGill University as well as the Research Committee of the Cree Board of Health and Social Services of James Bay (CBHSSJB).

### RBC FA determination

Participants were asked to fast overnight for at least 8 h. Blood samples were then taken by a registered nurse using standard protocols (12). Whole blood and plasma were stored temporarily at −20°C. Thereafter, samples were shipped to Quebec City, where they were kept frozen at −80°C at the Research Centre of the Centre Hospitalier Universitaire (CHU). FAs were quantified in RBC membrane phospholipids by gas–liquid chromatography, as described by Zhou et al. (13) and were expressed as the percentage of total FAs. FA determination has been previously described elsewhere (14).

### Anthropometric, clinical, lifestyle data and dietary assessment

Anthropometric, clinical and lifestyle data were collected using an interviewer-administered questionnaire either in *Iiyiyiuyimuwin* or English to gather sociodemographic and lifestyle information, such as tobacco and alcohol consumption. Dietary intake was assessed using a semi-quantitative food frequency questionnaire (FFQ) of traditional foods, taking into account seasonal variations and store-bought food items, and a 24-h recall. Height was measured to the nearest centimetre. Weight was measured using a Tanita digital scale (Tanita Corp., Arlington Heights, IL). Waist circumference (WC) was assessed at the end of exhalation with an inelastic tape located midway between the last floating rib and the iliac crest. WC ≥88 cm in women and ≥102 cm in men were defined as abdominal obesity. The systolic pressure (SBP) and diastolic blood pressure (DBP) were assessed according to the Canadian Hypertension Education Program recommendations (15). SBP ≥140 mmHg indicated high SBP, and DBP ≥90 mmHg indicated high DBP. HBP indicated high SBP and/or high DBP at study time, or a previous diagnosis of HBP without medication in the medical file.

### Cardiometabolic risk factors assessment

Cholesterol and triacylglycerol (TG) levels were measured by enzymatic methods in a Vitros 950 Chemistry Station (Ortho-Clinical Diagnostics, Raritan, NJ). HDL-cholesterol (HDL-c) was assessed directly with a Roche HDL-c plus third-generation reagent. LDL-cholesterol (LDL-c) was calculated by Friedewald's formula. Apolipoproteins (apo) A1 and B-100 were quantified by nephelometry in a BN ProSpec station (Dade Behring, Mississauga, ON). Fasting plasma glucose was measured enzymatically and fasting plasma insulin concentrations were measured with a commercial double-antibody radioimmunoassay (RIA) as described by Dewailly et al. (16). The homeostasis model assessment of insulin resistance (HOMA-IR) was calculated using the formula fasting plasma glucose (mmol/l)×fasting plasma insulin (*m*U/l)/22.5.

### Statistical analysis

Arithmetic means and standard deviation (SD) were reported for comparison with other studies. Geometric mean and 95% CI was also reported for transformed variables. The Chi-square test was used to analyze categorical variables, and the Student's *t*-test for continuous variables. Adjusted means for FAs between age categories (18–29, 30–39, 40–49 and 50–74 years) were obtained by an analysis of covariance (ANCOVA), with the Bonferroni correction for multiple comparisons (p<0.008), and were adjusted for gender and area of residence. Tests for the trend across age categories were assessed using the SAS software PROC GLM CONTRAST. Correlations between selected FAs were evaluated with Pearson's correlation coefficient (r). Insulin followed a non-normal residual distribution in the models and was then log transformed. Associations between RBC n-3 LC-PUFA and cardiometabolic risk factors (dependent variables) were examined by multiple linear regression analysis. Model 1 was adjusted for core sociodemographic factors, that is, age (continuous), gender, area of residence (coastal/inland), WC (continuous), smoking status (never/former/occasional/regular smoker), alcohol consumption (continuous) and blood mercury (continuous). Model 2 was further adjusted for α-linolenic acid (ALA, 18:3n3) (continuous). Model 3 was further adjusted for total n-6 PUFAs (continuous). Model 4 was further adjusted for monounsaturated FAs (MUFAs), saturated FAs (SFAs) and *trans* FAs (TFAs) (all continuous). The potential interaction between RBC n-3 LC-PUFAs, age and gender was also tested by regression modelling. No significant interactions were found between RBC n-3 LC-PUFAs and gender but interactions were significant (p<0.05) between RBC n-3 LC-PUFAs and age categories. Stratified regression analyses were also performed for model 4 by age categories. All analyses were performed with SAS software, version 9.2 (2008, SAS Institute Inc., Cary, NC. P values reported are two-sided (p<0.05).

## Results

The characteristics of the participants are shown in Table I. Th mean age was 38 years, the prevalence of overweight/obesity was 89% (with 69% obesity) and 52% were regular smokers. Most participants had total cholesterol (TC) and LDL-c concentrations within the normal range but 33% had hypertriglyceridemia, 44% had low plasma HDL-c and 77% had fasting plasma insulin ≥90 pmol/l, with women being more likely to be affected. Men had higher TC, LDL-c, SBP, DBP and apo B-100, while women had higher mean body mass index (BMI) and a prevalence of obesity, higher HDL-c, fasting insulin and HOMA-IR.

**Table I:** Characteristics of participants in *Eeyou Istchee* communities of Northern Québec

	Total (n=829)	Men (n=345)	Women (n=484)
Age (years)	38.2±14	38.9±14.3	37.7±13.8
18–29 (%)	30.6	29.9	31.2
30–39 (%)	29.1	27.3	30.4
40–49 (%)	18.7	19.7	18.0
50–74 (%)	21.6	23.2	20.5
BMI (kg/m^2^)	33.8±7.0	31.9±5.9	35.2±7.3*
<18.5 (%)	2.5	2.9	2.3*
18.5–25 (%)	8.2	11.9	5.6
25–30 (%)	20.4	24.6	17.4
≥30 (%)	68.9	60.6	74.8
WC (cm)	111.4±16	110.3±16	112.2±16
Men ≥102 cm, Women ≥88 cm (%)	84.7	71.9	93.8*
Smoking status (%)			
Never	9.5	9.6	9.4
Formerly	38.1	39.9	36.8
Occasionally (1–30 cigarettes/week)	12.6	12.0	13.0
Regular (2–25 cigarettes/day)	39.8	38.4	40.8
Never drinkers (%)	58.6	45.7	38.5*
TC (mmol/l)	4.6±0.9	4.8±0.9	4.5±0.9*
≥6.0 (%)	7.3	11.3	4.5*
LDL-c (mmol/l)	2.7±0.8	2.9±0.8	2.5±0.7*
≥4.5 (%)	2.0	3.4	1.1*
Apo B-100 (g/l)	1±0.4	1±0.4	0.9±0.4*
≥1.22 g/l (%)	23.3	26.5	21.0
HDL-c (mmol/l)	1.2±0.3	1.2±0.3	1.3±0.3*
Men <1.0, women <1.3 (%)	44.3	24.8	58.0*
Apo A1 (g/l)	1.3±0.4	1.3±0.3	1.3±0.4
<1.2 g/l (%)	30.9	32.3	30.0
TC: HDL	3.9±1.3	4.3±1.6	3.7±1*
≥6.0 (%)	4.6	7.7	2.4*
TG (mmol/l)	1.6±1.1	1.7±1.4	1.5±0.8
≥1.7 (%)	32.6	35.5	30.6
Fasting glucose (mmol/l)	6.3±2.5	6.2±2.2	6.4±2.7
Normal (<5.6) (%)	50.8	49.4	51.8
Impaired glucose (5.6–6.9) (%)	32.8	34.8	31.5
T2D (≥7.0) (%)	16.4	15.9	16.7
Fasting insulin (pmol/l)^a^	141 (136–148)	125 (117–134)	154 (147–162)
≥90 (%)	76.6	66.2	83.9*
HOMA-IR	48.1±47.6	42.2±37.1	52.3±53.4*
SBP (mm Hg)	121±15	125±14	119±15*
DBP (mm Hg)	74±11	77±10	72±11*
SBP ≥140 and/or DBP ≥90 (%)	14.5	17.3	12.5

Arithmetic mean±SD (all such values).

Apo: apolipoprotein; BMI: body mass index; DBP: diastolic blood pressure; HDL-c: HDL-cholesterol; HOMA-IR: homeostasis model assessment of insulin resistance; LDL-c: LDL-cholesterol; SBP: systolic blood pressure; T2D: type 2 diabetes; TC: total cholesterol; TG: triacylglycerols; WC: waist circumference.

* P<0.05 derived from two-sided *t*-test for continuous variables and Chi-square test for categorical variables.

a Geometric mean (95% CI).

Table II summarizes the RBC FA profile according to gender. Total n-3 PUFAs accounted for 6% of total FAs, with 96% represented by n-3 LC-PUFAs. At the same time, n-6 PUFAs accounted for 31% of total FAs and 83% of total PUFAs, with arachidonic acid (AA) contributing the most to n-6 PUFAs (47%). Except for DHA and linoleic acid (LA), FAs were similar between men and women. N-3 LC-PUFAs were inversely correlated with total n-6 PUFAs (r=−0.58, p<0.001), total SFAs (r=−0.10, p=0.003), total MUFAs (r=−0.15, p<0.001) and total TFAs (r=−0.10, p=0.003) (data not shown).

**Table II:** Fatty acid profiles in red blood cells in *Eeyou Istchee* communities of Northern Québec, according to gender

FA (% of total FAs)	Total (n=829)	Men (n=345)	Women (n=484)
Total PUFAs	37.2±1.4	37.2±1.4	37.2±1.5
Total n-3 PUFAs	6.3±1.3	6.3±1.4	6.3±1.3
ALA	0.13±0.12	0.19±0.12	0.14±0.12
EPA	0.51±0.24	0.53±0.27	0.50±0.22
DPAn-3	2.1±0.4	2.1±0.5	2.1±0.4
DHA	3.5±1.0	3.4±1.1	3.5±1.0*
n-3 LC-PUFA	6.1±1.3	6.0±1.4	6.1±1.2
Total n-6 PUFAs	30.9±1.7	31.0±1.7	30.9±1.7
LA	10.3±1.1	10.4±1.2	10.2±1.1*
AA	14.5±1.0	14.5±1.0	14.5±1.0
DPAn-6	0.81±0.15	0.80±0.15	0.81±0.16
SFAs	42.9±1.2	43.0±1.2	42.9±1.2
MUFAs	19.8±1.2	19.8±1.2	19.9±1.2
TFAs	0.67±0.51	0.63±0.51	0.70±0.52

Arithmetic mean±SD. *p<0.05.

AA: arachidonic acid (20:4n-6); ALA: α-linolenic acid (18:3n3); DHA: docosahexaenoic acid (22:6n-3); DPA: docosapentaenoic acid (22:5n-3 and 22:5n-6); EPA: eicosapentaenoic acid (20:5n-3); FA: fatty acid; LA: linoleic acid (18:2n-6); LC-PUFA: long chain-PUFA; MUFA: monounsaturated FA; n-3 LC-PUFA: EPA+DPAn-3+DHA; PUFA: polyunsaturated FA; SFA: saturated FA; TFA: *trans* FA.

Table III reports the RBC FA profiles according to age categories. Total n-3 PUFAs were higher among older participants, while total n-6 PUFAs were higher among younger participants (P_trend_<0.001). Total TFAs levels were lower with increased age, with RBC TFA being approximately 30% higher when comparing younger and older participants. About 40% of participants had mean RBC EPA+DHA ranging from 4 to 8% (of whom 82% were aged 50–74 years and 8% were aged 18–29 years) and 0.5% had a mean level over 8% (data not shown). At the same time, younger participants (aged 18–29 years) had ≈2 servings of fish per month, while the older age group (aged 50–74 years) had ≈2–3 servings per week (data not shown).

**Table III:** Fatty acid profiles in red blood cells in *Eeyou Istche*e communities of Northern Québec, according to age categories (n=829)

	Age categories				
		
FA (% of total FAs)	18–29 years (n=254)	30–39 years (n=241)	40–49 years (n=155)	50–74 years (n=179)	P_trend_
Total PUFAs	37.5±1.0^a^	37.1±1.6^a,b^	37.4±1.6^a,b^	36.9±1.5^b^	<0.001
Total n-3 PUFAs	5.6±0.8^a^	6.0±0.9^b^	6.6±1.4^c^	7.5±1.4^d^	<0.001
ALA	0.13±0.12	0.14±0.12	0.14±0.11	0.12±0.14	0.43
EPA	0.40±0.12^a^	0.46±0.15^b^	0.51±0.16^c^	0.76±0.34^d^	<0.001
DPAn-3	2.1±0.5^a,b^	2.1±0.4^a^	2.1±0.4^a,b^	2.2±0.3^b^	0.006
DHA	2.9±0.5^a^	3.2±0.7^b^	3.8±1.2^c^	4.4±1.0^d^	<0.001
n-3 LC-PUFA	5.4±0.8^a^	5.7±0.8^b^	6.3±1.3^c^	7.3±1.3^d^	<0.001
Total n-6 PUFAs	31.9±1.0^a^	31.2±1.5^b^	30.8±1.3^b^	29.4±1.9^c^	<0.001
LA	10.8±1.0^a^	10.4±0.8^b^	10.2±1.0^b^	9.4±1.3^c^	<0.001
AA	14.4±0.9	14.4±1.0	14.6±1.0	14.5±1.1	0.24
DPAn-6	0.85±0.12^a^	0.84±0.15^a,b^	0.80±0.14^b,c^	0.71±0.16^d^	<0.001
SFAs	42.7±0.9^a^	43.0±1.4^a,b^	43.0±1.2^a,b^	43.2±1.2^b,c^	<0.001
MUFAs	19.9±1.1	19.9±1.2	19.6±1.1	19.9±1.3	0.75
TFAs	0.75±0.53^a^	0.65±0.50^a,b^	0.67±0.48^a,b^	0.58±0.51^b,c^	0.001

Arithmetic mean±SD.

AA: arachidonic acid (20:4n-6); ALA: α-linolenic acid (18:3n3); DHA: docosahexaenoic acid (22:6n-3); DPA: docosapentaenoic acid (22:5n-3 and 22:5n-6); EPA: eicosapentaenoic acid (20:5n-3); FA: fatty acid; LA: linoleic acid (18:2n-6); LC-PUFA: long chain-PUFA; MUFA: monounsaturated FA; n-3 LC-PUFA; EPA+DPAn-3+DHA; PUFA: polyunsaturated FA; SFA: saturated FA; TFA: *trans* FA.

Different superscript letters indicate significant differences in mean values (ANCOVA with Bonferroni correction: p<0.008).

**Fig. 2:**
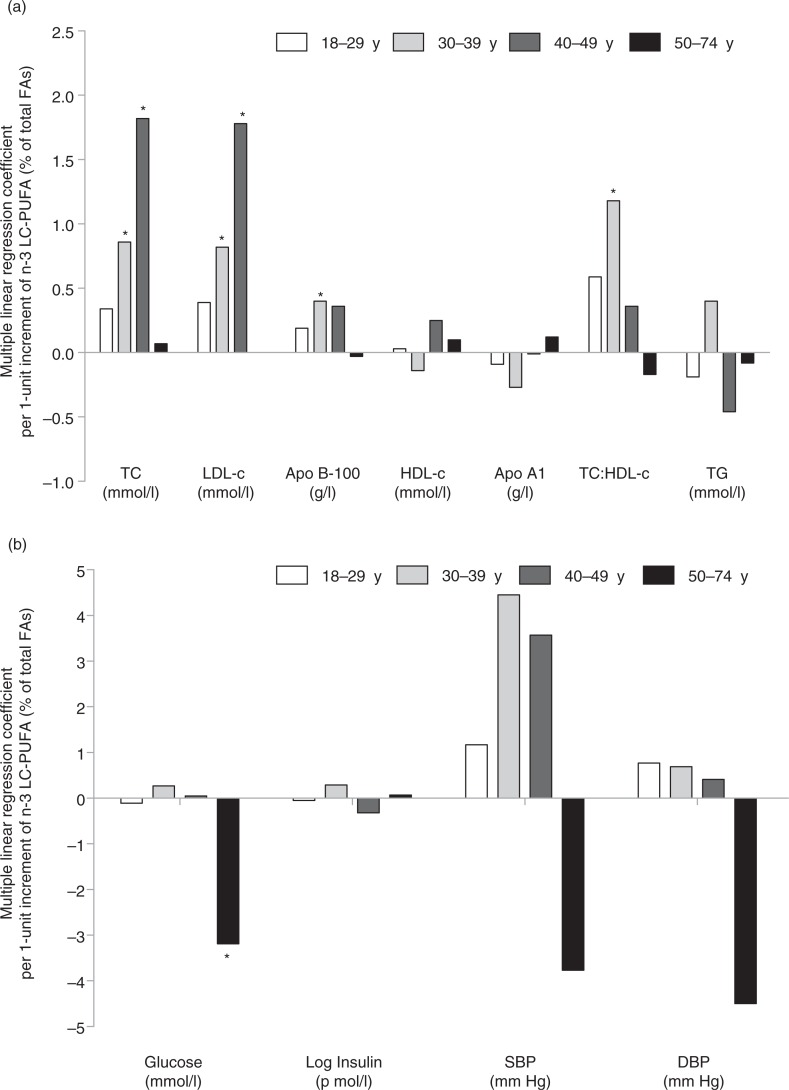
Multiple linear regression coefficients of selected cardiometabolic risk factors (dependent variables) per 1-unit increment of RBC n-3 LC-PUFA (predictor variable), according to age categories (n=613). The multivariate model was adjusted for age (continuous), gender, area of residence (coastal vs. inland), waist circumference (continuous), smoking status (never/former/occasionally/regular smokers), alcohol consumption (continuous), blood mercury level (continuous), ALA (continuous), total n-6 PUFAs (continuous), total SFAs, total MUFAs and total TFAs (all continuous). *p<0.05. ALA: α-linolenic acid (18:3n3); Apo: apolipoprotein; DBP: diastolic blood pressure; DHA: docosahexaenoic acid (22:6n-3); DPAn-3: docosapentaenoic acid (22:5n-3); EPA: eicosapentaenoic acid (20:5n-3); FA: fatty acid; HDL-c: HDL-cholesterol; LC-PUFA: long chain polyunsaturated FA; LDL-c: LDL-cholesterol; MUFA: monounsaturated FA; n-3 LC-PUFA: EPA+DPAn-3+DHA; SBP: systolic blood pressure; SFA: saturated FA; TC: total cholesterol; TFA: *trans* FA; TG: triacylglycerols.

After controlling for confounders in model 2, apo B-100 was the only significant cardiometabolic risk factor associated with RBC n-3 LC-PUFA (Table IV). However, after further adjustments for other FAs, RBC n-3 LC-PUFA was associated (p<0.05) with higher TC, LDL-c, apo B-100 and lower blood glucose (model 4). For TC and LDL-c, positive associations were noted among participants aged 30–39 years and 40–49 years but not among the younger (aged 18–29 years) or older (aged 50–74 years) participants, while positive associations for apo B-100 was noted only among those aged between 30 and 39 years (Fig. 2 and Table I). The association between RBC n-3 LC-PUFA and blood glucose was only statistically significant among older participants (aged 50–74 years). Details on the cardiometabolic risk factors by age categories among the Cree adults of our study are presented in the Supplementary File.

**Table IV:** Multiple linear regression coefficients of selected cardiometabolic risk factors (dependent variables) by the increment of RBC n-3 LC-PUFA (predictor variable) measured in Cree adults (n=613)

	Coefficients by the increment of RBC n-3 LC-PUFA							
	
Cardiometabolic risk factors	Model 1^a^		Model 2^b^		Model 3^c^		Model 4^d^	
				
	β	SEM	β	SEM	β	SEM	β	SEM
TC (mmol/l)	−0.003	0.03	−0.005	0.03	−0.02	0.04	0.57*	0.20
LDL-c (mmol/l)	0.0008	0.03	0.002	0.03	0.01	0.03	0.52*	0.17
Apo B-100 (g/l)	−0.03*	0.01	−0.03*	0.01	−0.04*	0.02	0.21*	0.07
HDL-c (mmol/l)	−0.008	0.01	−0.008	0.01	−0.005	0.01	0.04	0.07
Apo A1 (g/l)	0.02	0.01	0.02	0.01	0.02	0.01	−0.08	0.07
TC:HDL-c	0.03	0.04	0.02	0.04	0.01	0.04	0.42	0.24
TG (mmol/l)	0.006	0.03	−0.002	0.03	−0.04	0.03	0.02	0.17
Fasting glucose (mmol/l)	0.03	0.07	0.03	0.07	−0.01	0.07	−0.84*	0.40
Log fasting insulin (pmol/l)	0.05*	0.02	0.05	0.02	0.06*	0.02	0.03	0.11
SBP (mm Hg)	−0.27	0.52	−0.28	0.52	−0.40	0.57	−2.24	3.15
DBP (mm Hg)	0.11	0.42	0.10	0.43	−0.11	0.46	−1.98	2.55

* p<0.05.

ALA: α-linolenic acid (18:3n3); Apo: apolipoprotein; DBP: diastolic blood pressure; DHA: docosahexaenoic acid (22:6n-3); DPAn-3: docosapentaenoic acid (22:5n-3); EPA: eicosapentaenoic acid (20:5n-3); FA: fatty acid; HDL-c: HDL-cholesterol; LDL-c: LDL-cholesterol; MUFA: monounsaturated FA; LC-PUFA: long chain-PUFA; n-3 LC-PUFA: EPA+DPAn-3+DHA; PUFA: polyunsaturated FA; RBC: red blood cell; SBP: systolic blood pressure; SFA: saturated FA; TC: total cholesterol; TFA: *trans* FA; TG: triacylglycerols.

a Model 1 was adjusted for age (continuous), gender, area of residence (coastal vs. inland), waist circumference (continuous), smoking status (never/former/occasionally/regular smokers), alcohol consumption (continuous) and blood mercury level (continuous).

b Model 2 was further adjusted for ALA (continuous).

c Model 3 was further adjusted for total n-6 PUFAs (continuous).

d Model 4 was further adjusted for total SFAs, total MUFAs and total TFAs (all continuous).

## Discussion

In this adult, predominantly overweight/obese population of Cree living in Northern Québec, RBC n-3 LC-PUFAs, surrogate biomarkers of fat consumption from fish were not associated with a better cardiometabolic profile. Indeed, RBC n-3 LC-PUFAs were significantly associated with higher cardiometabolic risk in middle-age adults (aged 30–39 years and 40–49 years), and especially with higher TC, LDL-c and apo B-100. However, we observed that RBC n-3 LC-PUFAs were associated with lower blood glucose among older participants (aged 50–74 years).

No association was found between RBC n-3 LC-PUFA and HDL-c or TG levels. These results do not agree with previous studies in which n-3 PUFA intake (from fish or marine foods) was associated with an improvement of HDL-c and TG (higher HDL-c levels and lower TG) (17–19). Unlike the present study, in which FAs were measured in RBC, EPA+DHA were measured in plasma in the James Bay Cree adults in the 1991 Santé Québec Health Survey (19). However, other FAs were not included in statistical analyses. Moreover, RBC n-3 LC-PUFA was not associated with apo A1 (the major apolipoprotein in HDL-c particles). The inverse relationship between RBC n-3 LC-PUFA and apo A1 (Fig. 2 and Table I) was only noted in the 30–39 year age group (where n-3 levels were not the highest). Our observations indicate that RBC n-3 PUFA levels measured in the current study are insufficient to show a clinically significant association with plasma HDL-c or TG levels. This supports the theory that high n-3 PUFA intake (at least 1 g/day) is needed to significantly lower plasma TG (20).

RBC n-3 LC-PUFA was significantly associated with higher TC, LDL-c and apo B-100. Concentrations of apo B-100 (the primary apolipoprotein of LDL-c responsible for carrying cholesterol to tissues (21)) are related to coronary artery disease risk. However, Cree participants in the present study had mean apo B-100 (0.97 g/l) indicating a low risk (<1.04 g/l) according to Connelly et al. (22). Among the 917 James Bay Cree adults aged 18–74 years who participated in the 1991 Health Survey, EPA+DHA measured in plasma phospholipids were linked to higher levels of TC, LDL-c, SBP and DBP (19). Our positive association in participants aged 30–39 and 40–49 years, but not among the younger (aged 18–29 years) or older (aged 50–74 years) participants, is in line with 2 recent reviews of randomized controlled trials of n-3 LC-PUFAs (EPA and DHA) supplementation, in which DHA supplements were associated with a significantly higher LDL-c (23,24). A recent study among the Inuit from Nunavik (Northern Québec, Canada) also showed a positive association between a traditional dietary pattern and LDL-c concentrations that was attributed to a higher consumption of n-3 LC-PUFAs (25). This aligns with the fact that the nutritional transition is more pronounced in younger individuals (5).

With the exception of glucose among older participants (aged 50–74 years), our results did not show any beneficial effect of n-3 LC-PUFA on plasma fasting glucose and insulin, which supports the results of other studies in Northern communities (17–19). However, the relationship between n-3 PUFAs and glucose, insulin and T2D is not clear and is still controversial. Some observational studies found a protective action of certain types of n-3 LC-PUFAs on impaired plasma glucose or diabetes and insulin resistance in circumpolar populations (26,27), while a meta-analysis of randomized controlled trials found no beneficial effect on glycaemia and insulinaemia (28). According to studies in the early 1990s (10,19), when compared with the Nunavik Inuit surveyed in a similar time frame, Cree adults had much higher cardiometabolic risk factors (low HDL-c, high TG, high TC:HDL-c, high insulin and metabolic syndrome), and also had 1.7 times lower plasma n-3 LC-PUFA levels (10,18). More recent studies in the mid-2000s indicated that Cree adults had higher cardiometabolic risk factors (higher BMI, WC, SBP, TG, insulin, glucose) and lower HDL-c than the Inuit (29). This suggests that n-3 PUFAs levels observed might not be high enough among Cree to produce a beneficial effect on cardiometabolic risk factors. Obesity and T2D have increased dramatically in the Cree population and have become diseases of major importance in this population (30). Hyperinsulinaemia affected >75% of adults in the present study, and T2D afflicted ≈25% of adults.

In the present study, RBC n-3 LC-PUFA had no protective effect on DBP or SBP. This is similar to the results from previous studies of the James Bay Cree (14,19) and Nunavik Inuit (18) populations. Moreover, the Cree participants in our study had normal DBP/SBP measures (mean: 74/120 mmHg), which could explain the lack of association noted. Other studies among normotensive subjects also failed to show the effect of n-3 LC-PUFA on BP (31).

The RBC FA levels observed in our study are lower than those noted among Inuit (EPA=1.64, DPA=2.15, DHA=5.37, total n-3=9.49) (7). They are similar or slightly lower than those seen in US women (EPA=1.15, DPA=1.85, DHA=3.71, total n-3=7.06) (32). Moreover, we noted that RBC n-3 LC-PUFA was 1.4 times higher in older participants, which could be attributed to a higher intake of traditional foods, such as fish, that was observed in this study. Earlier studies have indeed shown that n-3 LC-PUFAs are positively correlated with fish intake (7,33) and are therefore considered valuable biomarkers of fish consumption. Thus, younger adults consumed less fish and other traditional foods than their parents. This nutritional transition is also ongoing in many other populations (34). As previously observed in other native peoples of North America (35–37), the nutritional transition appears to be particularly advanced among Cree, reflected by the shift away from traditional lifestyle reflected by a diet of store-bought foods and a sedentary lifestyle. This last aspect is associated with higher rates of CVD risk factors such as T2D, obesity or HBP (38).

One of the study's strengths is the analysis of biological FA measurements. Biological measurements of FA have the advantage of being able to characterize, objectively, long-term intake as opposed to dietary intakes assessed by 24-h recall questionnaires, in addition to reducing the risk of recall bias common in dietary questionnaires. Moreover, there were no missing values for the FA measurements. However, misclassification due to laboratory error cannot be excluded. Nevertheless, this study has some limitations due to its cross-sectional design, which does not permit us to ascertain any causal relationship. In our study, FAs are reported as a percentage by weight of the total FAs. We acknowledge that this is another limitation. Analytical methods developed for FA analysis have been primarily based on weight percentage calculations, in which each FA analyzed has an influence on the relative percentage of the other FAs rather than using weight or molar concentration (mg/l or mmol/l) measurements. Unfortunately, studies have shown that concentration-based methods do not always ensure more accurate results than weight percentage methods but do ensure easier interpretation of the results (39).

In conclusion, changing dietary habits among young Cree adults, reflected in the FA composition measured in their RBCs, may explain, at least partially, the extremely high prevalence of cardiometabolic risk factors, such as obesity, impaired glucose tolerance and hyperinsulinaemia in the present study, particularly among women. Although n-3 LC-PUFAs from traditional diet are known to have beneficial effects on cardiometabolic risk factors (9), the levels in Cree subjects might be insufficient to reverse these metabolic perturbations, especially if we consider the prevalence of cardiometabolic risk factors in this population. Overall dietary patterns are more important to a healthy diet than individual nutrients (40). The focus should be on the quality of the diet and the identification of its determinants. Finally, due to its cultural importance and good nutritional value, fish intake should continue to be encouraged. The consumption of fish provides the Cree population not only with n-3 LC-PUFA but also with physical, economic, sociocultural and spiritual benefits.

## Supplementary Material

Omega-3 polyunsaturated fatty acid profiles and relationship with cardiometabolic risk factors in Cree (*Eeyouch*) of Northern Québec
