# Supplementary material for: Omega-3 polyunsaturated fatty acid profiles and relationship with cardiometabolic risk factors in Cree (Eeyouch) of Northern Québec
Source: Int J Circumpolar Health. 2016 Jul 15;75:10.3402/ijch.v75.30361. doi: 10.3402/ijch.v75.30361 (PMC4947832; doi:10.3402/ijch.v75.30361)
Supplement: Omega-3 polyunsaturated fatty acid profiles and relationship with cardiometabolic risk factors in Cree (Eeyouch) of Northern Québec [file IJCH-75-30361-s001.docx]

**SUPPLEMENTARY TABLE**

Cardiometabolic risk factors in *Eeyou Istche*e communities of northern Québec, according to age categories (n=829).

| **Age categories** | | | | | |
| --- | --- | --- | --- | --- | --- |
| **Cardiometabolic** | **18–29 y**  (n=254) | **30–39 y**  (n=241) | **40–49 y**  (n=155) | **50–74 y**  (n=179) |  |
| **risk factors** |  |  |  |  | *P*_trend_ |
| TC (mmol/l) | 4.3±0.8^a^ | 4.7±0.8^bd^  2.8±0.7^bd^  1.0±0.4^b^  1.2±0.3^a^  1.3±0.3  4.1±1.1^ab^  1.7±1.0^b^  6.0±1.9^ab^ | 5.0±0.9^c^  3.0±0.8^c^  1.0±0.3^b^  1.2±0.3^ab^  1.4±0.3  4.2±1.1  1.7±0.9^b^  6.7±2.8^c^ | 4.7±1.0^d^  2.6±0.8^d^  1.1±0.4^b^  1.3±0.3^b^  1.3±0.4  3.8±1.7  1.7±1.7^ab^  7.7±3.3^d^ | <.0001 |
| LDL-c (mmol/l) | 2.4±0.7^a^  0.9±0.4^a^  1.2±0.3^a^  1.2±0.3  3.7±1.1  1.4±0.8^a^  5.4±1.4^a^ |  |  |  | <.0001 |
| Apo B-100 (g/l) |  |  |  |  | <.0001 |
| HDL-c (mmol/l) |  |  |  |  | 0.03 |
| Apo A1 (g/l) |  |  |  |  | 0.23 |
| TC:HDL-c |  |  |  |  | 0.34 |
| TG (mmol/l) |  |  |  |  | 0.03 |
| Fasting glucose (mmol/l) |  |  |  |  | <.0001 |
| Fasting insulin (pmol/l)^1^ | 131.8^a^  (122.0–142.5) | 142.7^ab^  (132.9–153.3) | 133.1^a^  (121.0–146.5) | 161.6^b^  (149.4–174.8) | 0.003 |
| SBP (mm Hg) | 71.1±11.1^a^  115.9±11.8^a^ | 75.8±11.0^b^  120.0±14.5^b^ | 76.6±9.5^b^  121.2±13.0^b^ | 74.2±11.1^c^  130.6±17.3^b^ | <.0001 |
| DBP (mm Hg) |  |  |  |  | 0.002 |

^1^Geometric mean (95% CI).

Arithmetic mean ± SD.

Apo: apolipoprotein; DBP: diastolic blood pressure; HDL-c: HDL-cholesterol; LDL-c: LDL-cholesterol; SBP: systolic blood pressure; TC: total cholesterol; TG: triacylglycerols.

Different superscript letters indicate significant differences in mean values (ANCOVA with Bonferroni correction: *P*<0.008).
